# Antibacterial Activity Prediction Model of Traditional Chinese Medicine Based on Combined Data-Driven Approach and Machine Learning Algorithm: Constructed and Validated

**DOI:** 10.3389/fmicb.2021.763498

**Published:** 2021-11-22

**Authors:** Jin-Tong Li, Ya-Wen Wei, Meng-Yu Wang, Chun-Xiao Yan, Xia Ren, Xian-Jun Fu

**Affiliations:** ^1^Institute of Traditional Chinese Medicine Literature and Culture, Shandong University of Traditional Chinese Medicine, Jinan, China; ^2^Marine Traditional Chinese Medicine Research Center, Shandong University of Traditional Chinese Medicine, Qingdao Academy of Traditional Chinese Medical Science, Qingdao, China; ^3^College of Pharmacy, Shandong University of Traditional Chinese Medicine, Jinan, China; ^4^Shandong Engineering and Technology Research Center of Traditional Chinese Medicine, Jinan, China

**Keywords:** traditional Chinese medicine (TCM), antibacterial activity, distribution law, machine learning, model construction

## Abstract

Traditional Chinese medicines (TCMs), as a unique natural medicine resource, were used to prevent and treat bacterial diseases in China with a long history. To provide a prediction model of screening antibacterial TCMs for the design and discovery of novel antibacterial agents, the literature about antibacterial TCMs in the China National Knowledge Infrastructure (CNKI) and Web of Science database was retrieved. The data were extracted and standardized. A total of 28,786 pieces of data from 904 antibacterial TCMs were collected. The data of plant medicine were the most numerous. The result of association rules mining showed a high correlation between antibacterial activity with cold nature, bitter and sour tastes, hemostatic, and purging fire efficacies. Moreover, TCMs with antibacterial activity showed a specific aggregation in the phylogenetic tree; 92% of them came from Tracheophyta, of which 74% were mainly concentrated in rosids, asterids, Liliopsida, and Ranunculales. The prediction models of anti-*Escherichia coli* and anti-*Staphylococcus aureus* activity, with AUC values (the area under the ROC curve) of 77.5 and 80.0%, respectively, were constructed by the Neural Networks (NN) algorithm after Bagged Classification and Regression Tree (Bagged CART) and Linear Discriminant Analysis (LDA) selection. The *in vitro* experimental results showed the prediction accuracy of these two models was 75 and 60%, respectively. Four TCMs (Cirsii Japonici Herba Carbonisata, Changii Radix, Swertiae Herba, Callicarpae Formosanae Folium) were proposed for the first time to show antibacterial activity against *E. coli* and/or *S. aureus*. The results implied that the prediction model of antibacterial activity of TCMs based on properties and families showed certain prediction ability, which was of great significance to the screening of antibacterial TCMs and can be used to discover novel antibacterial agents.

## Introduction

Bacterial diseases remain the primary cause of morbidity and mortality worldwide. Currently, antibiotics are effective drugs for the treatment of infections. In recent years, the abuse of antibacterial agents has led to the emergence of “superbacteria,” which has become an increasingly severe global public health problem (Deng et al., 2015). It is estimated that 10 million people will die from infections caused by drug-resistant per year by 2050 if the current situation is without intervention. The resulting economic losses will exceed 1.5 times of today’s world GDP (O’Neill, 2014). Moreover, antibacterial drugs kill pathogenic bacteria and may damage or inhibit normal bacteria at the same time, which causes bacterial flora disorder, so that the human body’s resistance is reduced (Cheng et al., 2017). Some antibiotics have been reported to alter the interactions between mitochondria and lysosomes, leading to apoptosis (Prajapati et al., 2019). Even some antibacterial drugs can cause rare side effects such as hypoglycemia, bone marrow suppression, and hyponatremia. In addition, studies have shown that a declining trend in antimicrobial resistance and the current space of bacterial resistance exceeds that of the speed of the development of new drug research (Schäberle and Hack, 2014; Lewis, 2020). Therefore, it is of great significance to develop new classes of antibacterial drugs.

Traditional Chinese medicines (TCMs), as a unique natural medicine resource in China, have a long history in the prevention and treatment of infectious diseases. Compared with other antibiotics, TCMs have the characteristics of being multi-component and multi-target, and potentially could be used to overcome drug resistance. Studies have shown that many TCMs, such as Coptidis Rhizoma and Lonicerae Japonicae Flos, have broad-spectrum antibacterial activity and different inhibitory degrees on gram-negative and gram-positive bacteria (Su et al., 2020). So, it has become a hot research topic to excavate antimicrobial drugs from TCMs over recent years. At present, experimental methods are mainly used to determine whether the TCMs have antibacterial activity. The long-observed cycle, high cost, and blind selection of Chinese medicines increase the difficulty of screening antibacterial TCMs.

With the era of big data coming, data mining technology and machine learning algorithms provide an innovative research direction for exploring TCMs. Data mining is a technique to find the rules from a large amount of data by analyzing every piece of data. The machine learning techniques are considered powerful tools to generate predictive models useful in virtual screening campaigns (Serafim et al., 2020). These methods have been widely used in the field of TCMs research. For example, data mining methods such as association rules have played an essential role in exploring and analyzing the rules of prescription formation and properties of TCMs (Fu et al., 2019; Shi et al., 2020). Furthermore, some scholars have applied machine learning in the fields of TCMs, such as toxicity prediction, tongue diagnosis, meridians prediction, and so on, providing a new idea for TCMs research (Liu et al., 2019; Wang et al., 2019; Wen et al., 2020). As to antibacterial research, Stokes et al. (2020) applied the neural network model to screen antibacterial components, which also provides a new approach for screening antibacterial drugs. Compared with the method based on *in vivo* experiments, the drug screening method based on machine learning and extensive data mining has become a potentially powerful technology due to its advantages of fast speed, low cost, etc.

The theory of medicinal properties is the core of TCMs, which mainly includes four natures, five tastes, meridian tropism, etc. It reflects the nature, performance, and clinical practice law of TCMs, contains clues to pharmacological effects, and has a great value to guide the clinical application of TCMs (Zhang et al., 2017). Our team used the association rules mining method to determine a specific correlation between the “Nature-Family-Component” of TCMs (Fu et al., 2013). In 2005, Academician Chen et al. (2005) also proposed a special relationship between “Family-Component-Efficacy” of the plant. The above results show a close relationship between property, family, component, and efficacy of TCMs. Based on this, our team has used co-occurrence analysis, molecular docking, chemical informatics, and other methods in the early stage to analyze the possible influencing factors such as the composition and structure skeleton of antibacterial TCMs (Wang et al., 2020).

**FIGURE 1:**
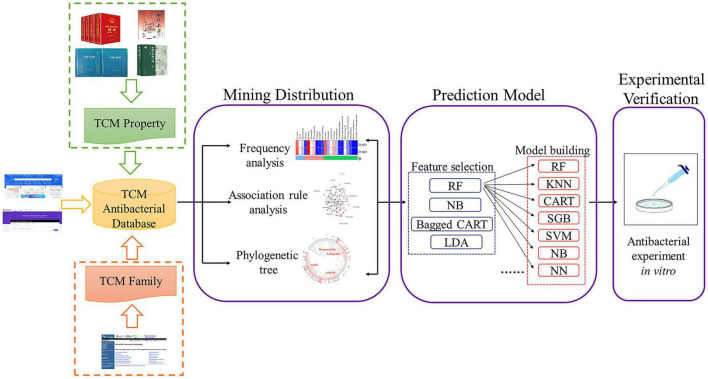
The whole framework of this study.

This study systematically sorted out the TCMs antibacterial literature published in China National Knowledge Infrastructure (CNKI) and Web of Science in the past 40 years. We mined the distribution law of properties, families, and species space of antibacterial TCMs through frequency analysis, association rules mining, and phylogenetic tree construction. Then, with the experimental information obtained from the antibacterial literature, the prediction models of antibacterial activity of TCMs against *Escherichia coli* (*E. coli*) and *Staphylococcus aureus* (*S. aureus*) were constructed by the machine algorithm, and the reliability was tested by antibacterial experiment. Our results provide a theoretical basis and essential data for improving the screening efficiency of antibacterial activity of TCM and the development and clinical practice of innovative antibacterial (Figure 1).

## Materials and Methods

### Data Sources and Screening Criteria

CNKI and Web of Sciences databases were selected as the database in this paper. The former used “(SU = “antibacterial” + “bacteriostatic”) and (KY = “traditional Chinese medicine” + “Chinese herbs”)” as search formula, and the latter used “TS = (antibacterial) and TS = (Chinese medicine or Chinese herbs or traditional Chinese medicine)” as the search formula. The search time was set from January 1979 to May 2021. Among them, candidate literature’s screening criteria included as follows: (1) Antibacterial or bacteriostatic experimental papers with clear antibacterial or bacteriostatic indicators, such as minimal inhibit concentration or inhibition zone; (2) Papers whose research object was TCM. According to the above criteria, we screened out the literature and the original data finally.

### Data Preprocessing

Firstly, the source of TCMs, four natures, five tastes, meridians tropism, efficacies and indications, the families, etc., were all imported into the table to form the original data. Secondly, the data was standardized, which consists of four parts: (1) The names of TCMs were standardized based on *Pharmacopeia of the People’s Republic of China* (Pharmacopoeia Commission China, 2020), *Chinese Materia Medica* (National Chinese Medicine Authority, 1999), *Grand Dictionary of Chinese Traditional Medicine* (Nanjing University of Chinese Medicine, 2006) and *National Compendium of Chinese Herbal Medicine* (Xie, 1976); (2) The four natures were standardized, and called “cool,” “slightly cold,” and “big cold” as cold, and “warm,” “slightly warm” and “big hot” referred to as hot; (3) The efficacy was standardized according to *Efficacy of Traditional Chinese Medicine* (Zhang, 2013); (4) Strain names were standardized based on *Bergey’s Manual of Determinative Bacteriology* (Buchanan and Gibbons, 1984), and the bacteria divided into gram-negative bacteria and gram-positive bacteria according to the gram staining information. Finally, the data that TCMs are mineral medicines or whose families are not clear was excluded.

### Data Analysis Methods

#### Frequency Analysis

The antibacterial TCMs were counted according to the families and their properties using Excel, and then the data was imported into Rstudio1.3.959 and was further visualized using the ggplot2 package.

#### Association Rule Mining

The information was imported into the R software platform, taking the families, properties, and efficacies of TCMs as the former item and the antibacterial effectiveness as the last item. Programs and the packages such as arules and arulesViz were used to mine and visualize the association rules (Hahsler, 2017). The correlation between the antibacterial activity of TCMs and the families, properties, and efficacies of TCMs was evaluated with the value of support, confidence, and lift of each rule.

#### Phylogenetic Tree Construction

The Taxonomy ID of TCMs original species was searched in the Taxonomy Database and uploaded to the Phylot website^1^ to generate a text file of the phylogenetic tree. And then, the text file was uploaded to Interactive tree of life (ITOL)^2^ for visual analysis (Ivica and Peer, 2011). In the phylogenetic tree, the antibacterial activity of the corresponding TCMs basic source species was marked. Finally, the distribution law of antibacterial TCMs in the phylogenetic tree of the basic source species was analyzed.

### Establishment of Machine Learning Model

#### Modeling Data Preprocessing

The data was imported into RStudio1.3.959 for preprocessing, which mainly included five parts: (1) Data integrity check: data with incomplete information was deleted. (2) Data encoding: the principle of “1 represents yes, 0 represents no” was used to describe the characteristics of TCMs properties, and the sequence 1,2,3.n was used to record the families’ characteristics. (3) Data type conversion: the dependent variables were transformed into the factor variables suitable for the classification model. (4) Data Splitting: the dataset was randomly split into 80% training data and 20% test data; (5) Data standardization and centralization: the data was standardized and centralized with the preProcess function.

#### Feature Selection Method

This paper uses the recursive feature elimination method for feature selection, which combines the feature selection process with the training process. The model’s predictive ability was used to measure feature selection (Chen et al., 2009). Random Forest (RF), Naive Bayes (NB), Bagged Classification and Regression Tree (Bagged CART), and Linear Discriminant Analysis (LDA) were fitted using the rfe function in the caret package, and 10-fold cross-validation was used in each process.

#### Establishment of Traditional Chinese Medicines Antibacterial Activity Prediction Model

After feature selection, six machine learning algorithms, namely Random Forest (RF), k-Nearest Neighbor (KNN), Classification and Regression Tree (CART), Support Vector Machine (SVM), Naive Bayes (NB), and Neural Networks (NN), were used to build models for different datasets. The train function implemented this process in the caret package, and five times 10-fold cross-validation was used in each model.

#### Evaluation of Traditional Chinese Medicines Antibacterial Activity Prediction Model

The receiver operator characteristic curve (ROC) of the model was established to predict the occurrence probability of the TCMs antibacterial activity, and calculating the area under the curve (AUC), comparing the performance and evaluating the predictive ability of each model (Baicus et al., 2011).

#### Optimization of Traditional Chinese Medicines Antibacterial Activity Prediction Model

The parameters of the screened model were optimized to improve the performance further. To obtain the optimal model, the parameters combinations of all models were traversed through the combination of grid parameter adjustment method and random parameter adjustment method. Among them, the critical parameter that significantly affects the KNN algorithm was the neighbor number k. The NN algorithm’s parameters needed to adjust were the number of hidden units size and the weight attenuation parameter decay.

### *In vitro* Antibacterial Tests of the Traditional Chinese Medicines

#### Reagents

Seventy five percent ethanol was purchased from Sinopharm Chemical Reagent Co., Ltd. (Shanghai, China), and Luria-Bertani (LB) Culture was purchased from Shanghai Aladdin Biochemical Technology Co., Ltd. (Shanghai, China).

#### Preparation of Traditional Chinese Medicines Extract

Dichroae Radix, Cirsii Japonici Herba Carbonisata, Swertiae Herba, Bubali Cornu, Callicarpae Formosanae Folium, Turpiniae Folium, Changii Radix, and Trachelospermi Caulis et Folium were all purchased from Qingdao Tiancheng Chinese Medicine Co., Ltd. (Shandong, China). The TCMs were identified by Professor Baoguo Li, Shandong University of Chinese Medicine.

Preparation of ethanol extract of TCMs: Firstly, 50 g of TCM samples were crushed and soaked 10 times in 75% ethanol for 1 h, followed by reflux extraction at 100°C for 2 h. Secondly, the extract was collected, and the remaining residue was extracted eight times using 75% ethanol at 80°C for 1 h. Finally, the two sections were mixed and vacuumed filtrated, and the filtrate was concentrated by rotary evaporation at 45°C to obtain 1 g/mL ethanol extract and stored in –20°C refrigerator until use.

Preparation of water extract of TCMs: 75% ethanol was replaced with pure water. According to the preparation method of alcohol extract, the water extract with 1 g/mL was obtained.

#### Preparation of Bacterial Strains

*E. coli* and *S. aureus* were certified and provided by the Ocean University of China and were stored at –20°C. Firstly, the bacteria were activated before use, and 15–20 mL LB culture was poured into the sterilized Petri dish to be spread evenly and solidified for use. Secondly, the inoculation loop was used to dip the bacterial solution, and a “Z” line was drawn on the solid medium. Then, after being rotated 90°, a second line was drawn in the same way, and the operated Petri dishes were sealed with sealing strips and incubated at 37°C for 24 h. Finally, the bacteria were picked up by an inoculation loop in sterile water, and the bacterial solution was shaken until even. Compared with the shaken McFarland Turbidity standard tube, the bacterial suspension containing about 1 × 106 ∼ 1 × 107 CFU⋅mL^–1^ was prepared and placed for reserve.

#### Antibacterial Activity Assay

A total of 100 μL of the prepared *E. coli* suspension were absorbed and evenly spread on the Petri dishes containing 15–20 mL LB culture. Four holes were drilled in the agar medium orderly of each dish. Different TCM extracts (100 μL) were added to the holes of each Petri dish. The water extract was added to the up and down spots, and the ethanol extract was added to the left and right holes. Finally, the inoculated plates were sealed with sealing strips, labeled corresponding information, and incubated for 24 h at 37°C in a constant temperature biochemical incubator. The production of a bacteriostatic ring was observed, and the diameter of the bacteriostatic ring (mm) was measured with a vernier caliper. The experiment was repeated three times.

## Results

### Sources of Antibacterial Traditional Chinese Medicines

**FIGURE 2:**
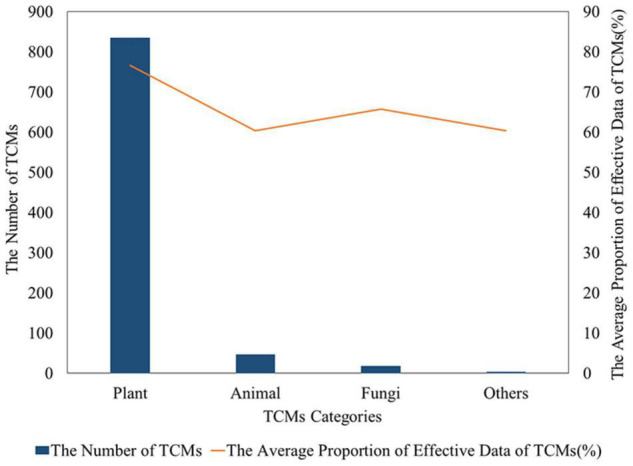
Distribution of antibacterial TCMs from different sources.

According to the screening criteria, 904 TCMs were included in the analysis from 999 original species. The number of TCMs from plants (835) was the largest, accounting for 92.4% of the total number, followed by animal TCMs and fungi TCMs. According to the average proportion of the number of effective TCMs from different species, the plant TCMs were the highest, followed by fungi TCMs, animal TCMs, and others (Figure 2).

### Distribution Law of Antibacterial Traditional Chinese Medicines Properties

#### Frequency Analysis of Traditional Chinese Medicines Properties

**FIGURE 3:**
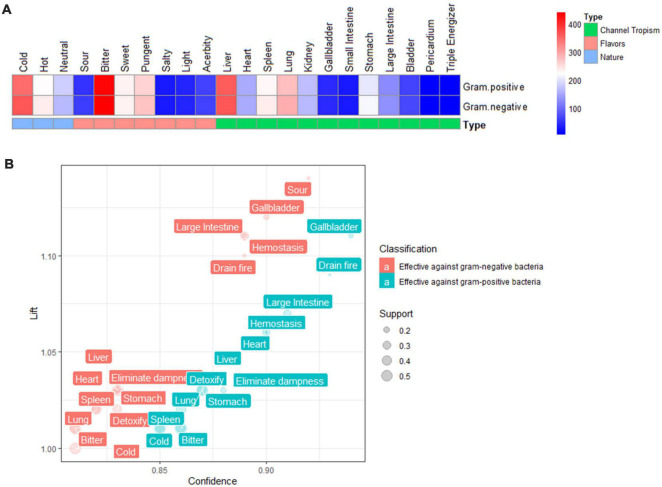
Distribution law of antibacterial TCMs properties. **(A)** Heatmap of antibacterial TCMs properties frequency. **(B)** Comparison results of association rules for the properties of TCM against gram-positive bacteria and gram-negative bacteria. Cold is the medicinal nature; bitter and sour are the medicinal tastes; the liver, heart, spleen, lung, gallbladder, stomach, and large intestine are the meridians tropism; hemostasis, drain fire, eliminate dampness, and detoxify are medicinal efficacies.

The frequency analysis results on the properties of antibacterial TCMs showed that most medicines were cold nature, bitter, pungent and sweet tastes. Most of the meridians tropism were liver, lung, spleen, and stomach. The statistical results of the two kinds of antibacterial TCMs properties are the same (Figure 3A).

#### Association Rule Analysis of Traditional Chinese Medicines Properties

Association rules are an essential technology of data mining to mine the correlation between valuable data from a large amount of data, reflecting the interdependence and correlation between things (Tung et al., 2003). The association rules between TCMs properties and anti-gram-positive bacteria and anti-gram-positive bacteria were explored (support > 10%, confidence > 80%). The results showed a strong relationship between cold TCMs and the anti-gram-negative bacteria and anti-gram-positive bacteria activity, and the confidence degree was 81 and 85%, respectively. In terms of taste, sour TCMs had a higher degree of confidence (about 92%) with effective resistance to gram-negative bacteria, followed by bitter TCMs with a confidence of 81%. However, among the effective rules against gram-positive bacteria, bitter TCMs had the highest correlation degree with the confidence of 86%. Among the meridian tropism rules meeting the threshold, the most effective correlation with gram-negative bacteria was the gallbladder meridian (90%), followed by the large intestine meridian (89%), liver meridian (83%), heart meridian (83%), stomach meridian (83%), lung meridian (82%), and spleen meridian (82%), in order of confidence. In terms of gram-positive bacteria, the gallbladder meridian (94%), large intestine meridian (91%), heart meridian (87%), liver meridian (87%), stomach meridian (86%), lung meridian (86%), spleen meridian (85%) had the order of confidence from high to low. As for the efficacy of TCMs, the hemostatic efficacy showed a higher correlation with the confidence of 89 and 90%, respectively. Following purging fire efficacy with the confidence of effectiveness against gram negative bacteria and gram-positive bacteria were 89%, 93%, Respectively (Figure 3B).

### Distribution Law of Antibacterial Traditional Chinese Medicines Families

#### Frequency Analysis of Traditional Chinese Medicines Families

**FIGURE 4:**
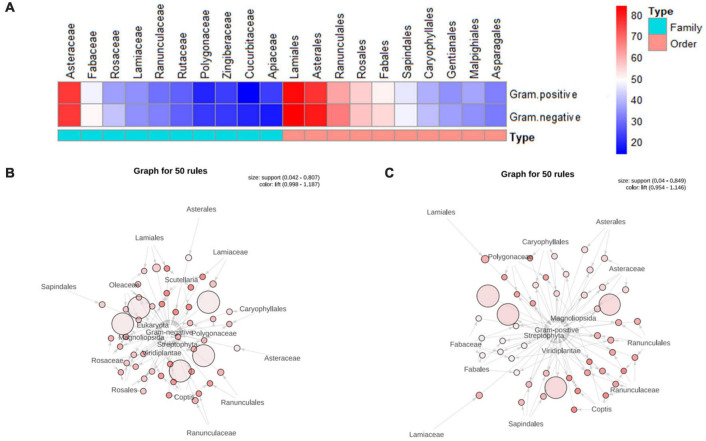
Distribution law of antibacterial TCMs families. **(A)** Heatmap of antibacterial TCMs orders and families frequency (TOP 10). **(B)** The association network of TCMs families against gram-negative bacteria. **(C)** The association network of TCMs families against gram-positive bacteria. Note: the circle in the figure represents association rules. The size of the circle represents the degree of support, the larger the circle, the greater the degree of support. The color of the circle represents the degree of confidence, the darker the color, the greater the degree of confidence. The arrow indicates the relationship between two things. The side pointing to the circle is the antecedent of the rule, and the side pointed out from the circle is the consequent of the rule.

According to statistics, 714 TCMs from 820 original species were effective against gram-negative bacteria, which were distributed in 170 families, 82 orders, 19 classes, and 9 phyla. Among them, the most significant number of species were found in Asteraceae (76), and Fabaceae (50), Rosaceae (41), Lamiaceae (34), and Ranunculaceae (32) with more than 30 species. The most numerous orders were Lamiales (84), followed by Asterales (80), Ranunculales (67), Rosales (57), and Fabales (54). A total of 730 TCMs were effective against gram-positive bacteria, from 832 original species, distributed in 179 families, 86 orders, 20 classes, and 9 phyla. Similar to the statistical results of TCMs against gram-negative bacteria, the top three families with the largest number of species were Asteraceae (76), Fabaceae (47), and Rosaceae (36), and the top three orders with the largest number of species were Lamiales (82), Asterales (77) and Ranunculales (61) (Figure 4A).

#### Association Rule Analysis of Traditional Chinese Medicines Families

A total of 147 rules for the families of antibacterial TCMs and the activity against Gram-negative bacteria were screened (support > 4%, confidence > 60%). The results showed that the highest compelling correlation with anti-gram-negative bacteria activity was Ranunculaceae and Rosaceae, with the confidence of about 91%, followed by Polygonaceae with the confidence of about 90%. The rest were Lamiaceae, Oleaceae, and Asteraceae, which had more than 80% confidence. The orders with high effective associations with anti-gram-negative bacteria activity were Ranunculales, Rosales, Lamiales, Sapindales, and Caryophyllales (Figure 4B). With the same support and confidence as thresholds, the rules of TCMs families and anti-gram-positive bacteria activity were excavated. Polygonaceae, Ranunculaceae, Lamiaceae, Asteraceae, and Fabaceae were the top five families with the highest effective correlation against anti-gram-positive bacteria activity. The order with the highest confidence was Ranunculales (93%), followed by Lamiales, Sapindales, and Asterales (Figure 4C).

#### Distribution of Antibacterial Traditional Chinese Medicines’ Original Species on the Phylogenetic Tree

To further study the distribution of antimicrobial TCMs’ original species in the phylogenetic tree, all TCMs in *Pharmacopoeia of the People’s Republic of China* (Pharmacopoeia Commission China, 2020), *Chinese Materia Medica* (National Chinese Medicine Authority, 1999), *Grand Dictionary of Chinese Traditional Medicine* (Nanjing University of Chinese Medicine, 2006) and *National Compendium of Chinese Herbal Medicine* (Xie, 1976) were collected. The Chinese mineral medicines and some TCMs without Taxonomy ID were eliminated. There were 11,272 TCMs from 742 families. ITOL was used to construct the distribution of TCMs with antibacterial activity in the phylogenetic tree of the 11,272 TCMs original species. The end nodes of the branches represented families of different species, and species with similar genetic relationships will also be close in the tree. In the outer ring, the anti-gram-negative bacteria activity and anti-gram-positive bacteria activity were indicated in blue and red, respectively.

**FIGURE 5:**
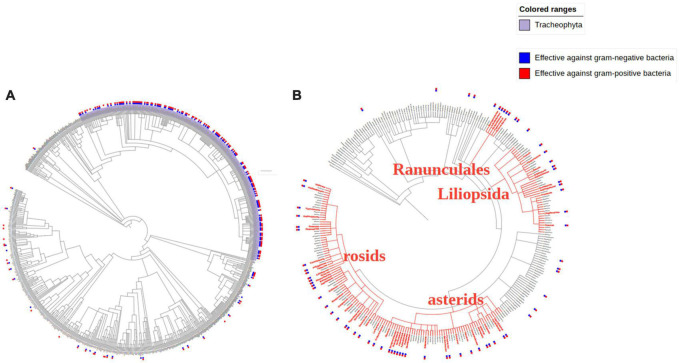
Distribution of antibacterial TCMs original species on the phylogenetic tree **(A)** and Distribution of antibacterial TCMs in phylogenetic tree of Viridiplantae kingdom **(B)**.

The results showed that 187 families had produced TCMs with antibacterial activity and showed specific aggregation. Most TCMs with antibacterial activity came from Tracheophyta (about 92% of all antibacterial TCMs). The rest were scattered in Metazoa, Fungi, and other kingdoms, consistent with the previous results of the mass of the number of plant TCMs (Figure 5A).

In the phylogenetic tree of the Viridiplantae kingdom, families with more than two species were identified in the figure due to the significant difference in the number of species in antibacterial TCMs families. The results demonstrated a specific aggregation of TCMs, mainly concentrated in the four branches of rosids, asterids, Liliopsida, and Ranunculales. The antibacterial TCMs in these four branches accounted for 74% of all plant antibacterial TCMs (Figure 5B).

### Bacterial Species Statistics

**FIGURE 6:**
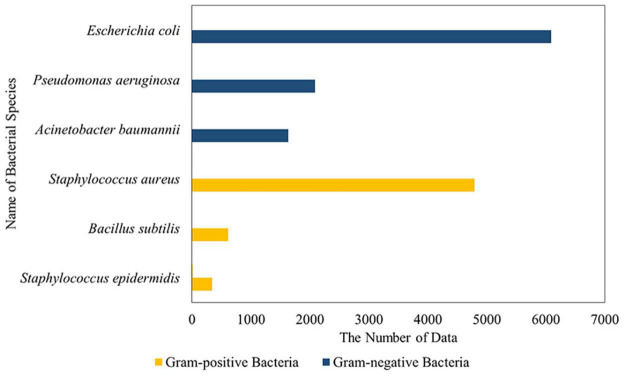
Statistical chart of bacterial species (TOP 3).

According to the statistical results, 19,083 anti-gram-negative bacteria and 9,703 anti-gram-positive bacteria data were found. The largest gram-negative bacteria were *E. coli* with 6093, followed by *Pseudomonas aeruginosa* (2086) and *Acinetobacter baumannii* (1634). The most significant number of gram-positive bacteria was *S. aureus* (4790), followed by *Bacillus subtilis* (641) and *Staphylococcus epidermis* (343) (Figure 6). *E. coli* and *S. aureus* were representative strains of gram-negative bacteria and gram-positive bacteria, respectively. Therefore, *E. coli* and *S. aureus* were selected as the data of the two prediction models, respectively.

### Prediction Models of Antibacterial Activity of Traditional Chinese Medicines

The above analysis of the antibacterial TCMs properties and families found that the TCMs with antibacterial activity had certain regularity and characteristics on the distribution of properties and families. Consequently, the natures (cold, hot, neutral), tastes (sour, bitter, sweet, pungent, salty, light, acerbity), meridians tropism (liver, heart, spleen, lung, kidney, gallbladder, small intestine, stomach, large intestine, bladder, pericardium, triple energizer), and families (kingdom, phylum, class, order, family, and genus) were taken as the characteristics to construct prediction model of antibacterial activity.

#### Feature Selection of Prediction Models for Antibacterial Activity of Traditional Chinese Medicines

**FIGURE 7:**
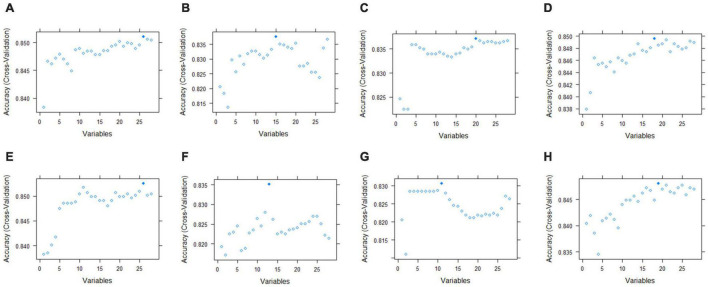
Results of feature selection of models. **(A–D)** Represented fitting RF, NB, LDA and Bagged CART functions, respectively, and standed for anti-*E. coli* model results. **(E–H)** Represented fitting RF, NB, LDA, and Bagged CART functions, respectively, and standed for anti- *S. aureus* model results.

Feature selection is an effective method to reduce the number of features in input data. Too much redundancy often increases the calculation overhead and hurts the model (Li et al., 2017). The recursive feature elimination method can remove features with low weight coefficients through continuous training to select high-quality variable subsets. Therefore, we applied the recursive feature elimination method to fit four RF, NB, LDA, and Bagged CART functions for feature selection. In the feature selection of the anti-*E. coli* model, 26 features were retained after RF selection, with an accuracy of 85.1% for cross-validation; 15 features were included after NB selection, with an accuracy of 83.8%; and 20 components were retained after LDA selection, with an accuracy of 83.7%. After Bagged CART selection, 18 features were kept, and the accuracy was 85.0% (Figures 7A–D and Supplementary Table 1). The feature selection results of the anti-*S. aureus* model were similar to those of the anti-*E. coli* model. The number of features retained after RF function feature selection was the largest, and the cross-validation accuracy was also high. After selection, 26 features were maintained with an accuracy of 85.3%. After selection by NB, LDA, and Bagged CART, 15, 20, and 18 components were retained, with accuracy of 83.5, 83.1, and 84.8%, respectively (Figures 7E–H and Supplementary Table 1). In this way, the number of features in the input data was reduced, and the model’s performance was improved.

#### Algorithm Selection for Prediction Models of Antibacterial Activity of Traditional Chinese Medicines

**FIGURE 8:**
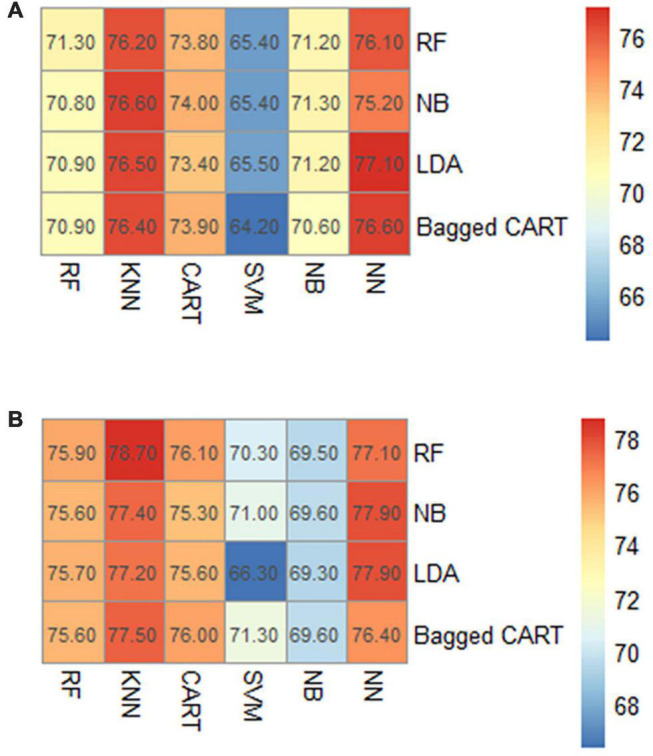
AUC results of different algorithms. **(A)** Anti-*E. coli* model. **(B)** Anti-*S. aureus* model.

This paper selected six machine learning algorithms, RF, KNN, CART, SVM, NB, and NN, in the prediction model of TCMs against *E. coli* and *S. aureus*. We evaluated the model performance by comparing the AUC value. According to the AUC results of the prediction model of anti-*E. coli* activity, the KNN and NN algorithms had better performance with the AUC value reaching more than 75%. The CART algorithm was slightly inferior to the above algorithms, the RF and NB algorithms had similar results, and the SVM algorithm had the worst performance (Figure 8A). In the prediction model of anti-*S. aureus* activity, RF, KNN, CART, and NN were the algorithms with AUC values above 75%, respectively, among which KNN and NN had the best performance. On the contrary, SVM and NB performed worst in this model (Figure 8B).

#### Algorithm Optimization for Prediction Models of Antibacterial Activity of Traditional Chinese Medicines

**FIGURE 9:**
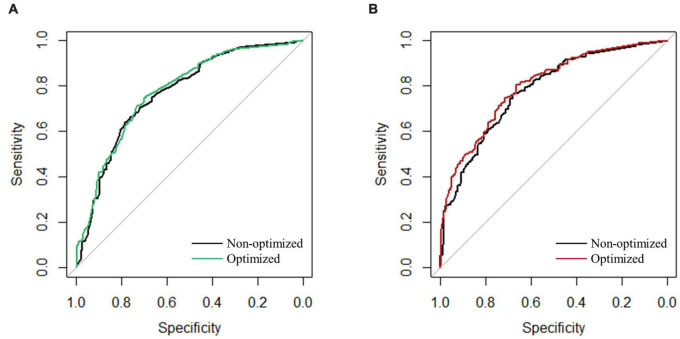
ROC curve before and after optimization. **(A)** Anti-*E. coli* model (NN model after Bagged CART selection). **(B)** Anti-*S. aureus* model (NN model after LDA selection).

The model parameters were adjusted to achieve the optimal parameter combination to reduce the loss in the test set and improve the prediction accuracy. The optimization results were shown in the AUC value improvement. The top five algorithms of each model were selected from the AUC results of the antibacterial prediction models for optimization. In the prediction model of anti-*E. coli* activity, the NN algorithm was selected after LDA and Bagged CART, and KNN algorithms selected by NB, LDA, and KNN, were chosen for optimization. After optimization, the AUC value of each algorithm increased by varying degrees. Among them, the NN after Bagged CART selection had the best results, with the AUC value rising from 76.6 to 77.5% (Table 1 and Figure 9A). In the prediction model of anti-*S. aureus* activity, the NN algorithms selected by NB and LDA, and KNN algorithms, chosen by RF, Bagged CART, and NB, were optimized, respectively. After LDA selection, the results showed that NN performed best with the most significant increase, whose AUC value increased from 77.9 to 80.0% (Table 1 and Figure 9B).

**TABLE 1:** AUC value optimization of antibacterial activity prediction models.

Model	Algorithms	Non-optimized AUC value (%)	Optimized AUC value (%)
Anti-*E. coli* model	NN model after LDA selection	77.1	77.4
	NN model after Bagged CART selection	76.6	77.5
	KNN model after NB selection	76.6	76.8
	KNN model after LDA selection	76.5	76.7
	KNN model after Bagged CART selection	76.4	76.9
Anti-*S. aureus* model	KNN model after RF selection	78.7	78.8
	NN model after NB selection	77.9	78.6
	NN model after LDA selection	77.9	80.0
	KNN model after Bagged CART selection	77.5	77.6
	KNN model after NB selection	77.4	78.3

#### Application of Prediction Models for Antibacterial Activity of Traditional Chinese Medicines

The above models were applied to the screening of antibacterial TCMs. To predict the anti-*E. coli* anti-*S. aureus* activity, some TCMs were sorted out from *Pharmacopoeia of the People’s Republic of China* (Pharmacopoeia Commission China, 2020). The screening criteria were as follows:(1) TCMs with a bitter taste, cold nature, and return to the liver meridian; (2) excluding TCMs with antibacterial activity already reported in the literature; (3) excluding Chinese mineral medicines and TCMs with unclear source information. The prediction model predicted the antibacterial activity of the sorted TCMs, and the results were shown in the table (Supplementary Table 2). In the prediction results of the anti-*E. coli* activity, Cirsii Japonici Herba Carbonisata, and Swertiae Herba were effective, while six TCMs including Dichroae Radix and Turpiniae Folium were not. In the prediction results of anti-*S. aureus* activity, however, five TCMs, including Dichroae Radix and Cirsii Japonici Herba Carbonisata, were effective (Table 2).

**TABLE 2:** Prediction results of TCMs antibacterial activity.

Scientific name	Resistance to *E. coli*	Resistance to *S. aureus*
*Dichroa febrifuga* Lour.	F	T
*Cirsium japonicum* Fisch.ex DC.	T	T
*Swertia pseudochinensis* Hara	T	T
*Bubalus bubalis* Linnaeus	F	T
*Callicarpa formosana* Rolfe	F	T
*Turpinia arguta* Seem.	F	–
*Changium smyrnioides* Wolff	F	–
*Trachelospermum jasminoides* (Lindl.) Lem.	F	–

*T stands for valid; F stands for invalid; “–” indicates that the corresponding TCMs and strain do not meet the screening conditions.*

### Validation of the Prediction Using Antibacterial Experiments *in vitro*

**FIGURE 10:**
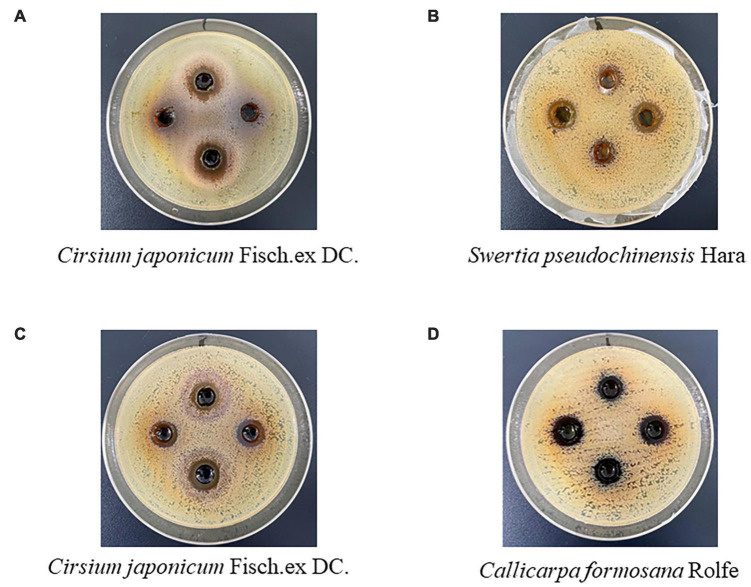
Inhibition zone of TCMs extract against *E. coli* and *S. aureus*. **(A)** Inhibition zone of TCMs extract on *E. coli*. **(B–D)** Inhibition zone of TCMs extract on *S. aureus*. The water extract was added to the up and down holes, and the ethanol extract was added to the left and right holes.

To validate the accuracy of the prediction model, we tested the antibacterial activity of the above TCMs *in vitro*. In this paper, the antibacterial activity of TCMs to be tested by the bacteriostatic circle method. The bacteriostatic circle method uses medicines to inhibit the growth of bacteria around it and form a transparent circle. It is a classic method widely used to determine the antibacterial activity of drugs due to its convenient operation, low cost, accurate and reliable results. If the water or ethanol extract of these TCMs had a bacteriostatic circle, it was regarded as effective; otherwise, it was regarded as invalid. The results showed that Cirsii Japonici Herba Carbonisata and Changii Radix had inhibitory effects on *E. coli* (Table 3 and Figure 10A). Cirsii Japonici Herba Carbonisata, Swertiae Herba, Callicarpae Formosanae Folium were effective on *S. aureus* (Table 3 and Figures 10B–D).

**TABLE 3:** Inhibition zone of TCMs extract on *E. coli* and *S. aureus* (mm).

Bacterial strains	Scientific name	Water extract	Ethanol extract	Activity
*E. coli*	*Dichroa febrifuga* Lour.	0	0	F
	*Cirsium japonicum* Fisch.ex DC.	14.75 ± 1.30	0	T
	*Swertia pseudochinensis* Hara	0	0	F
	*Bubalus bubalis* Linnaeus	0	0	F
	*Callicarpa formosana* Rolfe	0	0	F
	*Turpinia arguta* Seem.	0	0	F
	*Changium smyrnioides* Wolff	9.00 ± 0.10	0	T
	*Trachelospermum jasminoides* (Lindl.) Lem.	0	0	F
*S. aureus*	*Dichroa febrifuga* Lour.	0	0	F
	*Cirsium japonicum* Fisch.ex DC.	11.75 ± 2.78	11.50 ± 2.06	T
	*Swertia pseudochinensis* Hara	11.25 ± 0.83	12.75 ± 1.30	T
	*Bubalus bubalis* Linnaeus	0	0	F
	*Callicarpa formosana* Rolfe	12.00 ± 0.71	13.75 ± 0.43	T

*T, stands for valid; F, stands for invalid.*

### Results Comparison Between Models’ Prediction and *in vitro* Experiments

After comparing the results between models’ prediction and *in vitro* experimental, it is known that in TCMs activity of *E. coli* model prediction, the predicted effects of the model results did not agree with the experiment for two herbs (Swertiae Herba, Changii Radix) and the predicted six TCMs, including Dichroae Radix and Cirsii Japonici Herba Carbonisata, were in accordance with the experimental results. Overall the accuracy of prediction was 75%. In the forecast of the anti-*S. aureus* activity of TCMs, the results of Dichroae Radix and Bubali Cornu were inconsistent with the experiment; however, three TCMs (Cirsii Japonici Herba Carbonisata, Swertiae Herba, Callicarpae Formosanae Folium) were in good agreement with the experiment. The prediction accuracy was 60% (Table 4).

**TABLE 4:** Comparison between models’ prediction results and experimental results.

Scientific name	Comparison between prediction results and experimental results	
	Resistance to *E. coli*	Resistance to *S. aureus*
*Dichroa febrifuga* Lour.	Y	N
*Cirsium japonicum* Fisch.ex DC.	Y	Y
*Swertia pseudochinensis* Hara	N	Y
*Bubalus bubalis* Linnaeus	Y	N
*Callicarpa formosana* Rolfe	Y	Y
*Turpinia arguta* Seem.	Y	–
*Changium smyrnioides* Wolff	N	–
*Trachelospermum jasminoides* (Lindl.) Lem.	Y	–

*Y means that the two results are consistent; N means that the two results are inconsistent; “–” indicates that the corresponding TCMs and strain do not meet the screening conditions.*

## Discussion

As a medical resource with unique advantages and core intellectual property rights, TCMs have become a significant research trend in mining antibacterial under the background of widespread bacterial infectious diseases. This study found that plant antibacterial TCMs were the hotspot of current research, and their antibacterial activity and species were different. For example, Ophiopogonis Radix, Platycladi Cacumen, and Artemisiae Argyi Folium are mainly resistant to gram-positive bacteria, while Pulsatillae Radix and Mori Cortex are primarily resistant to gram-negative bacteria (Huang et al., 2018). Although the numbers of fungi TCMs are small, their effectiveness is relatively high and has the potential for further development.

By excavating the distribution rules of properties of antibacterial TCMs, TCMs with cold nature and bitter, sweet and pungent taste accounted for the most significant proportion of antibacterial medicines. In association rule analysis, TCMs with cold nature or bitter taste showed a high correlation with anti-gram-negative bacteria and anti-gram-positive bacteria activity. The majority of TCMs with cold nature and bitter taste have the effect of clearing heat and draining fire, according to the medication principle of internal heat syndrome caused by a bacterial infection (Ma et al., 2019). Notably, although the proportion of sour TCMs is not high in antibacterial TCMs, they have the highest compelling correlation with anti-gram-negative bacteria activity due to the most significant number of *E. coli* in gram-negative bacteria data. *E. coli* can cause gastrointestinal infection in humans and various animals under certain conditions leading to diarrhea, while sour medicine is mainly used for astringency and diarrhea (Chen and Frankel, 2005; Zhong, 2016). In terms of the meridian tropism of antibacterial TCMs, our results suggested that the main meridians tropism of antibacterial TCM were the gallbladder, large intestine, liver, heart, stomach, lung, and spleen meridians. Meridian tropism closely associates the drug action with the viscera meridians of the human body and orientates the drug action to provide the basis for clinical dialectical drug-using (Zhong, 2016). As a result, in selecting antibacterial TCMs, the corresponding TCM can be chosen according to the disease lesion site caused by bacteria. For example, TCMs returning to the lung meridian can be reflected in treating respiratory tract infection, pharyngitis, and other diseases caused by bacteria (Deng and Zhang, 2019). As for the efficacy of TCMs, the effect of hemostasis was highly correlated with the impact of anti-gram-negative bacteria and anti-gram-positive bacteria activity, which was consistent with the anti-infection effect of almost all hemostatic TCMs, such as Sanguisorbae Radix, Sophorae Flos, and Cirsii Herba (Hao et al., 2005).

According to the TCMs origin species, TCMs in Asteraceae, Fabaceae, and Rosaceae showed antibacterial activity. Fabaceae had a higher effective correlation with anti-gram-positive bacteria activity, and Rosaceae had a higher effective correlation with anti-gram-negative bacteria activity. Ranunculaceae, Polygonaceae, Lamiaceae, and Asteraceae were highly correlated with anti-gram-negative bacteria and anti-gram-positive bacteria activity. For example, Coptidis Rhizoma in Ranunculaceae, with the reputation of “TCM antibiotics,” had better activity against gram-positive bacteria and gram-negative bacteria (Li et al., 2015). Polygonaceae, such as Rhei Radix et Rhizoma, can inhibit and kill bacteria in a certain concentration range before and after processing (Yang W. T. et al., 2020). Besides, Scutellariae Radix, a TCM of Lamiaceae, had a certain inhibitory effect on multiple bacterias (Li et al., 2015). Atractylodis rhizoma in Asteraceae also showed better antibacterial effects (He et al., 2020).

The phylogenetic tree is a way to describe the relationships between different organisms. Recently, research has shown that phylogenetic ties can be used to find the formation and development trends of a certain biological group and could be used as a prediction tool. Studies pointed out that the predictive function of the phylogenetic tree can be used to discover TCMs with the same biological activity or to analyze TCMs with similar efficacy (Fu et al., 2017). In this study, with the help of phylogenetic relationship analysis, we explored the distribution characteristics of antibacterial TCMs in species space. The result indicated that TCMs with antibacterial activity had a certain aggregation and focused on specific branches in the phylogenetic tree (rosids, asterids, Liliopsida, and Ranunculales). This brings us to an important point: the closer the species is to the TCMs with antibacterial activity, the closer the kinship is, and there is a greater possibility that they also have antibacterial activity, which provides ideas and reference for the discovery of new antibacterial TCMs.

The above analysis suggested that antibacterial TCMs had a certain law in medication properties and distribution of families. Based on this, we suggest that the characteristic collection can be constructed using TCM properties and families information. We used the algorithms to build a model to predict the antibacterial activity of TCMs. When it was hard to obtain the effective indexes to evaluate the antibacterial activity of TCMs directly, the results of antibacterial activity were reflected by the relevant information affecting its activity, which significantly reduced the trial and error cost compared with the traditional experimental methods (Arji et al., 2019). The recursive feature elimination method was used to fit different functions to reduce the dimension of the feature set. It was found that a relatively small number of features selected by Bagged CART could be retained, but the accuracy was relatively high. Although the cross-validation accuracy of the elements chosen by RF was the highest, the number of included features was also the largest. Too many features, on the contrary, could reduce the prediction ability of the model (Cai et al., 2018). Moreover, in the construction process of the two models, both the KNN and NN algorithms showed better performance, but the NN algorithms were selected in the final results. One of the reasons was that NN showed greater potential with the AUC value being improved by up to 2.1% after optimization. In terms of KNN algorithms, however, the AUC value could only increase by 0.9% at the highest after optimization. The NN algorithm does not impose any conditions on the input variables. It has an excellent ability to deal with the non-linear characteristics of TCMs, such as the four natures, five tastes, and meridians tropism (Tang et al., 2021). The optimized AUC values of the prediction model for anti-*E. coli* activity and for anti-*S. aureus* activity reached 77.5 and 80.0%, respectively. The model’s prediction accuracy was 75 and 60%, respectively, compared with the experimental results. It had a certain prediction ability for the antibacterial activity of TCMs, but there was still a gap with the ideal results. This may be because many factors were affecting the antibacterial activity and the current characteristics information was not comprehensive enough. In the future, relevant characteristics such as composition and target can be considered to be added (Wang et al., 2020).

In this paper, we firstly proposed that Cirsii Japonici Herba Carbonisata and Changii Radix had inhibitory effects on *E. coli* while Cirsii Japonici Herba Carbonisata, Swertiae Herba and Callicarpae Formosanae Folium were effective to *S. aureus*. The water extract of Cirsii Japonici Herba Carbonisata showed apparent inhibition on *E. coli*, and the ethanol extract of Callicarpae Formosanae Folium had the best bacteriostatic effect on *S. aureus*. Cirsii Japonici Herba Carbonisata is a processed product of Cirsii Japonici Herba. At present, Cirsii Japonici Herba Carbonisata has been reported to contain a large number of organic acids and flavonoids. Quercetin, as a representative component of flavonoids, has been proved to inhibit a variety of bacteria, including *E. coli* and *S. aureus* (Deng et al., 2009; Yang Y. et al., 2020). Changii Radix is derived from the root of the Codonopsis pilosula of *Platycodon grandiflorum*. It contains many chemical components, including polysaccharides, coumarins, sterols, unsaturated fatty acids, and other components. Studies have shown that vanillic acid, β-sitosterol, and stigmasterol in Changii Radix have an apparent inhibitory effect on *E. coli* (Ji et al., 2015; Ododo et al., 2016; Yusuf et al., 2018; Qian et al., 2020b). The ingredients of Swertiae Herba are complex, mainly including iridoids, ketones, alkaloids, and triterpenes. Among the ketones, bellidifolin has inhibitory effects on *S. aureus*, *E. coli*, *Salmonella*, and *Pseudomonas aeruginosa* (Lu et al., 2015; Duan et al., 2021). Callicarpae Formosanae Folium contains various antibacterial components, such as luteolin and apigenin, which can inhibit the growth of *S. aureus* to a certain extent (Cheng et al., 2018; Qian et al., 2020a). The crude extraction of TCMs cannot reveal the drug’s ability because of the complex composition of TCMs. These medicines will show more significant potential if the extraction process can be optimized, providing a reference for screening new antibacterial materials.

## Conclusion

This study analyzed the distribution of reported antibacterial TCMs based on frequency analysis and association rules. The results showed that the cold and bitter TCMs had good antibacterial activity. Furthermore, TCMs in rosids, asterids, Liliopsida, and Ranunculales had potential antibacterial TCM screening value. On this basis, the prediction models of anti-*E. coli* and anti-*S. aureus* activity were constructed with the characteristics of TCMs properties and families information. The prediction accuracy of the models was 75 and 60%, respectively. Using the constructed antibacterial model, we found four new antibacterial TCMs against *E. coli* and/or *S. aureus*. These models can predict the antibacterial activity of TCMs, which provides a scientific basis for the clinical application of TCMs antibiotics and the research and development of innovative drugs.

## Data Availability Statement

The original contributions presented in the study are included in the article/Supplementary Material, further inquiries can be directed to the corresponding author/s.

## Author Contributions

X-JF and XR conceived and designed the experiments, edited, and reviewed the manuscript. J-TL, Y-WW, M-YW, and C-XY performed the experiments. J-TL wrote the original draft. All authors have read, revised, and approved the final manuscript.

## Conflict of Interest

The authors declare that the research was conducted in the absence of any commercial or financial relationships that could be construed as a potential conflict of interest.

## Publisher’s Note

All claims expressed in this article are solely those of the authors and do not necessarily represent those of their affiliated organizations, or those of the publisher, the editors and the reviewers. Any product that may be evaluated in this article, or claim that may be made by its manufacturer, is not guaranteed or endorsed by the publisher.

## Footnotes

1
https://phylot.biobyte.de/

2
https://itol.embl.de/
