# Supplementary material for: Antibacterial Activity Prediction Model of Traditional Chinese Medicine Based on Combined Data-Driven Approach and Machine Learning Algorithm: Constructed and Validated
Source: Front Microbiol. 2021 Nov 22;12:763498. doi: 10.3389/fmicb.2021.763498 (PMC8645695; doi:10.3389/fmicb.2021.763498)
Supplement: Supplementary file 1 [file Data_Sheet_1.DOCX]

Supplementary Material

# Supplementary Tables

**Supplementary Table 1.** Retention characteristics of anti-*E.coli* model and anti-*S.aureus* model after feature selection

| Model | Feature Selection Fitting Function | Feature |
| --- | --- | --- |
| Anti-*E.coli* model | RF | Order; Genus; Family; Heart; Liver; Large Intestine; Cold; Pungent; Bitter; Sweet; Small Intestine; Lung; Kidney; Spleen; Stomach; Class; Hot; Sour; Gallbladder; Acerbity; Neutral; Bladder; Phylum; Light; Salty; Kingdom |
|  | NB | Order; Large Intestine; Family; Acerbity; Gallbladder; Heart; Liver; Sour; Lung; Spleen; Stomach; Sweet; Genus; Pungent; Class |
|  | LDA | Order; Large Intestine; Family; Acerbity; Gallbladder; Heart; Liver; Sour; Lung; Spleen; Stomach; Sweet; Genus; Pungent; Class; Cold; Hot; Phylum; Kingdom; Small Intestine |
|  | Bagged CART | Order; Family; Genus; Acerbity; Class; Large Intestine; Cold; Bitter; Neutral; Liver; Sweet; Heart; Spleen; Lung; Stomach; Pungent; Small Intestine; Kidney |
| Anti-*S.aureus* model | RF | Order; Genus; Family; Sweet; Lung; Neutral; Stomach; Liver; Hot; Cold; Pungent; Bitter; Heart; Large Intestine; Sour; Class; Gallbladder; Spleen; Bladder; Kidney; Phylum; Kingdom; Small Intestine; Salty; Triple Energizer; Acerbity |
|  | NB | Family; Order; Acerbity; Heart; Liver; Gallbladder; Large Intestine; Spleen; Stomach; Sweet; Genus; Small Intestine; Class |
|  | LDA | Family; Order; Acerbity; Heart; Liver; Gallbladder; Large Intestine; Spleen; Stomach; Sweet; Genus |
|  | Bagged CART | Order; Family; Genus; Acerbity; Class; Lung; Spleen; Liver; Cold; Sweet; Bitter; Hot; Heart; Stomach; Pungent; Neutral; Gallbladder; Kidney; Large Intestine |

**Supplementary Table 2.** TCMs selected for predicting

| Scientific name | Chinese Name | Latin Name^a^ | Parts used |
| --- | --- | --- | --- |
| *Dichroa febrifuga* Lour. | Changshan | Dichroae Radix | Root |
| *Cirsium japonicum* Fisch.ex DC. | Dajitan | Cirsii Japonici Herba Carbonisata | Stem;Leaf; Flower |
| *Swertia pseudochinensis* Hara | Dangyao | Swertiae Herba | Whole herb |
| *Bubalus bubalis* Linnaeus | Shuiniujiao | Bubali Cornu | Horn |
| *Callicarpa formosana* Rolfe | Zizhuye | Callicarpae Formosanae Folium | Leaf |
| *Turpinia arguta* Seem. | Shanxiangyuanye | Turpiniae Folium | Leaf |
| *Changium smyrnioides* Wolff | Mingdangshen | Changii Radix | Root |
| *Trachelospermum jasminoides*(Lindl.) Lem. | Luoshiteng | Trachelospermi Caulis et Folium | Stem |

a: The Latin names by Chinese Pharmacopoeia (2020 Edition)
